# Socioeconomic Disparity in Survival after Breast Cancer in Ireland: Observational Study

**DOI:** 10.1371/journal.pone.0111729

**Published:** 2014-11-05

**Authors:** Paul M. Walsh, Julianne Byrne, Maria Kelly, Joe McDevitt, Harry Comber

**Affiliations:** 1 National Cancer Registry, Cork, Ireland; 2 Boyne Research Institute, Drogheda, Ireland; University of Campinas, Brazil

## Abstract

We evaluated the relationship between breast cancer survival and deprivation using data from the Irish National Cancer Registry. Cause-specific survival was compared between five area-based socioeconomic deprivation strata using Cox regression. Patient and tumour characteristics and treatment were compared using modified Poisson regression with robust variance estimation. Based on 21356 patients diagnosed 1999–2008, age-standardized five-year survival averaged 80% in the least deprived and 75% in the most deprived stratum. Age-adjusted mortality risk was 33% higher in the most deprived group (hazard ratio 1.33, 95% CI 1.21–1.45, P<0.001). The most deprived groups were more likely to present with advanced stage, high grade or hormone receptor-negative cancer, symptomatically, or with significant comorbidity, and to be smokers or unmarried, and less likely to have breast-conserving surgery. Cox modelling suggested that the available data on patient, tumour and treatment factors could account for only about half of the survival disparity (adjusted hazard ratio 1.18, 95% CI 0.97–1.43, P = 0.093). Survival disparity did not diminish over time, compared with the period 1994–1998. Persistent survival disparities among Irish breast cancer patients suggest unequal use of or access to services and highlight the need for further research to understand and remove the behavioural or other barriers involved.

## Introduction

The incidence of invasive breast cancer in Irish women has increased steadily since national statistics were first collated in 1994. Incidence in Irish women was close to the average for European women in 2002, but has moved to the position of fourth from the top in recent years [1]. On average, 2,518 women were diagnosed with invasive breast cancer each year in the five years to 2009. However, the death rate has fallen markedly since the early 1990 s [1].

Factors that can adversely affect survival rates after breast cancer, including their relationship to socioeconomic deprivation, have been extensively studied elsewhere. They include, among others, comorbidity [2]–[5], region of residence [6], educational attainment [7]–[10], participation in screening programmes [4], [11]–[12], and diagnosis at a late stage [13]–[19]. Many of these studies, and a range of others, have noted socioeconomic disparity in breast cancer survival or in factors potentially influencing survival [20]–[31]. But on the whole there has been relatively little consensus as to the mechanisms or the importance of contributory or mediating factors involved, although stage at diagnosis and access to adequate treatment are widely seen as the most important.

We evaluated socioeconomic influences on breast cancer survival in Ireland using data collected by the National Cancer Registry. The objective was to quantify the survival disparities, and to identify relevant factors and attempt to quantify their role.

## Materials and Methods

### Study population and survival outcomes

Women diagnosed with invasive breast cancer in Ireland at ages 15–99 years during 1994–2008 were included. Patients who had the same date of diagnosis and death (mainly death-certificate-only and autopsy-only cases) or who had had a previous cancer (other than non-melanoma skin cancer) were excluded. The main focus was on the years 1999–2008, because quality and completeness of data on some factors (notably treatment and receptor status) was higher for those years. Survival follow-up was by matching against national death certificate data, complete to 31 December 2009. Cause-specific survival was the main outcome, and cause of death was assigned to breast cancer if coded as invasive or non-invasive breast tumour, cancer of unspecified thoracic site, or cancer of unspecified site [32].

### SAHRU deprivation index

We used the area-based SAHRU index of social deprivation [33]–[34], based on the 3409 electoral divisions in the Republic of Ireland. The index is constructed from five census indicators covering employment levels, social class, car ownership, home rental status, and overcrowding. Deprivation scores derived from data in the 2002 census at electoral division level were applied to individual patients by address linkage, and the ten SAHRU strata were re-grouped into five strata (1 = least deprived to 5 = most deprived). Note that these strata are not strict quintiles.

### Other patient and tumour variables

Age-groups 15–44, 45–54, 55–64, 65–74 and 75–99 were used for age-standardization and adjustment [35], but broader age-groups (15–49, 50–64 and 65–99) for some descriptive or summary purposes. Region of residence was defined by Health Service Executive (HSE) administrative areas – Dublin/Mid-Leinster, Dublin/North-East, South, and West. Smoking status was defined as current, former, never or unknown; marital status as currently married, divorced/separated, widowed, never married or unknown.

TNM 5th-edition stage (I to IV and unknown) was used for descriptive summaries [36], but fuller T, N and M categories of stage were used in models (all node-positive cases were combined; detailed T categories were used in models but combined for some descriptive purposes). Grade was coded as 1, 2, 3–4 or unknown; tumour morphology as ductal adenocarcinoma, lobular adenocarcinoma, other adenocarcinoma, other specific carcinoma, unspecified carcinoma, unspecified cancer, sarcoma, or malignant phyllodes tumour. HER2 status was defined as negative, ambiguous, positive, or unknown; hormone receptor status as negative, positive or unknown (based on highest available score for oestrogen or progesterone receptors). Method of presentation was defined as symptomatic, screening (population-based and other combined), incidental, or unknown.

Comorbidity was coded by linkage of cancer registry data for the years 2002–2008 to public hospital data (Hospital Inpatient Enquiry System) [37] to identify other significant medical conditions within a year following (or a month before) breast cancer diagnosis. The Charlson Index of comorbidity (0 = no significant conditions other than breast cancer, 1 = one other, or 2 = two or more other conditions) was derived for each linked patient [38]. Linkage was achieved for 13039 patients (82%) in those years, covering admissions (both publicly and private-insurance funded) in public hospitals.

### Treatment variables

Treatment data analysed were from the 12 months following diagnosis. The variables defined (yes/no unless indicated) were: lymph-node biopsy/excision; most advanced breast surgery (mastectomy, breast-conserving surgery, or none); radiotherapy; chemotherapy (including biological response modifiers and immunotherapy); and hormone therapy. Times to first treatment were also coded for each modality (as before median date, median date or later, or none, and as detailed month of treatment) and tested for inclusion in survival models. Hormonal data, often prescribed on an outpatient basis, were known to be incomplete (NCR unpublished), thus cautious interpretation of findings is needed. Radiotherapy data from private hospitals were slightly incomplete for the years 2006–2008, thus some analyses were restricted to 1999–2005.

### Statistical methods

All analyses were done in Stata 11. Patient and tumour characteristics and treatments were compared between deprivation strata using modified Poisson regression with robust variance estimation, generating relative risks (RRs), more appropriate than odds ratios for such analyses [39]. Five-year and ten-year cause-specific survival was estimated actuarially, and Cox modelling was used to derive mortality hazard ratios associated with deprivation stratum, adjusting for age and (in fuller models) other relevant patient, tumour and treatment variables. Age, stage and grade showed non-proportional hazards, thus Cox models were stratified for these variables. Likelihood-ratio tests (with P<0.05 threshold) were used to identify factors for inclusion in ‘full’ models. In the latter models, the precision of hazard ratio estimates was adjusted to control for possible within-region correlations. In addition to overall models, Cox models of deprivation effects (age-specific or age-adjusted) were also applied to specific patient subgroups for assessment of heterogeneity of effects. All available follow-up was used for survival models, except when comparing diagnosis cohorts (five-year survival used only).

### Ethics statement

The National Cancer Registry has permission under the Health (Provision of Information) Act 1997 to collect and hold data on all persons diagnosed with cancer in Ireland. The use of these data for research is covered by the Statutory Instrument which established the Registry Board in 1991. The Registry was given permission to access anonymised Hospital Inpatient Enquiry System data under the Health (Provision of Information) Act 1997; permission was given by the Department of Health and the Economic and Social Research Institute (ESRI) who were joint custodians of the data. All datasets were anonymised prior to analysis.

## Results

### Breast cancer incidence and patient numbers

**Figure 1:**
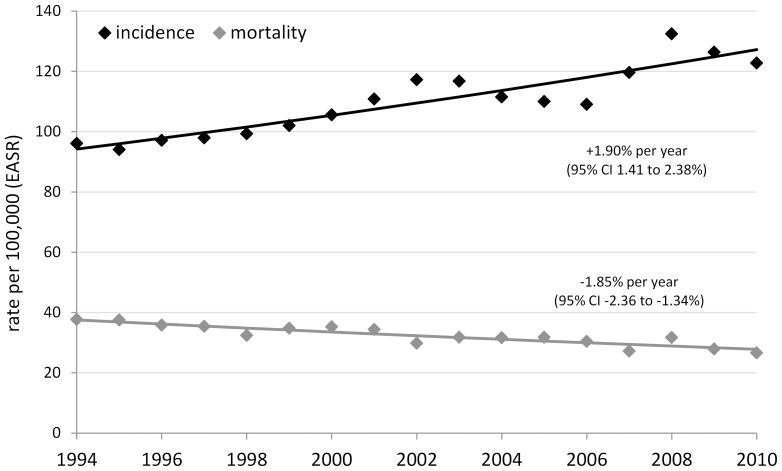
Trends in invasive breast cancer incidence and mortality, Ireland, 1994–2010 (European age-standardized rates).

Incidence rates of invasive breast cancer among Irish women increased by 1.9% per year, on average, while mortality rates fell by 1.8% per year between 1994 and 2010 (Figure 1). During 1999–2008, there were 22075 incident cases and the European age-standardized incidence rate averaged 113.8 cases per 100,000 (Table 1). Incidence showed a strong relationship to deprivation status, with rates 20% lower in the most deprived compared with the least deprived stratum (Table 1).

**Table 1:** Case numbers, incidence and summary of patient and tumour characteristics variables (those showing significant variation) by area-based deprivation stratum for female breast cancer in Ireland, 1999–2008.^a^

		Deprivation stratum (1 = least, 5 = most deprived)					^#^RR for deprivation	
	Total^f^	1	2	3	4	5	5 v 1	1–5 trend
Incident cases 1999–2008	22075	5295	2985	2812	3469	5688		
Incidence (EASR)^b^	113.8	–	–	–	–	–		
SIR (age-adjusted)^c^	–	1.00	0.86	0.87	0.82	0.80		
Cases for survival analysis^d^	21356	5160	2908	2723	3386	5517		
All deaths	5306	1105	685	690	861	1585		
Breast cancer deaths	3906	818	497	523	636	1137		
Person-years follow-up	99072	25011	13846	12522	15982	24996		
Median follow-up years	4.2	4.4	4.3	4.1	4.3	4.1		
Age 15–49 at diagnosis	25.6%	27.1%	28.6%	26.0%	24.9%	23.5%	***0.87	***0.96
Age 50–64 ““	40.1%	40.2%	40.2%	40.5%	39.6%	39.7%	0.99	1.00
Age 65–99 ““	34.3%	32.7%	31.2%	33.6%	35.5%	36.8%	***1.12	***1.03
Resident: Dublin/Mid-Leinster	29.9%	41.3%	31.4%	26.3%	19.0%	29.5%	***0.72	***0.90
Resident Dublin/North-East	20.8%	21.1%	24.4%	20.5%	18.2%	21.9%	1.04	0.99
Resident: South	25.8%	19.1%	26.7%	29.8%	34.0%	23.9%	***1.24	***1.06
Resident: West	23.4%	18.3%	17.4%	23.4%	28.7%	24.7%	***1.35	***1.10
Smoker: ever	30.5%	25.9%	28.6%	29.4%	31.3%	36.1%	***1.27	***1.06
Smoker: current	19.1%	14.3%	17.1%	17.1%	20.1%	25.1%	***1.62	***1.13
Married: ever	81.8%	82.5%	82.2%	83.1%	84.1%	78.0%	***0.94	***0.99
Married: current	59.4%	62.9%	63.1%	60.3%	59.7%	52.1%	***0.84	***0.96
Charlson Index 1 or 2^e^	10.9%	8.8%	9.8%	11.0%	11.2%	13.5%	***1.47	***1.09
Stage I	28.3%	31.1%	29.0%	26.7%	27.8%	26.7%	***0.86	***0.97
Stage II	46.5%	45.7%	47.4%	48.5%	46.0%	46.0%	1.01	1.00
Stage III	12.4%	11.3%	11.8%	12.8%	12.8%	13.5%	**1.18	***1.04
Stage IV	7.1%	6.0%	6.8%	6.9%	7.6%	8.0%	***1.29	***1.06
T1	38.9%	41.6%	40.2%	38.3%	37.3%	36.8%	***0.89	***0.97
T4	8.0%	6.4%	7.5%	7.5%	8.4%	9.4%	***1.39	***1.08
Regional node positive	41.5%	39.1%	42.2%	42.3%	41.8%	42.7%	**1.08	**1.02
Tumour grade 1	10.0%	11.1%	11.0%	8.8%	9.8%	9.4%	**0.84	**0.96
Tumour grade 3–4	32.2%	30.6%	31.7%	34.3%	31.5%	33.2%	**1.10	**1.02
Lobular adenocarcinoma	15.6%	16.6%	16.1%	14.9%	15.0%	15.4%	0.92	*0.98
HER2 negative	32.5%	32.7%	32.4%	34.8%	31.4%	30.9%	*0.95	*0.99
ER/PR positive	59.6%	61.2%	59.5%	59.0%	56.9%	58.9%	***0.96	***0.99
Presentation: symptoms	73.7%	67.9%	72.3%	74.8%	78.0%	76.5%	***1.04	***1.01
Presentation: screening	15.5%	17.2%	16.1%	15.6%	13.0%	14.9%	***0.83	***0.95

**Figure 2:**
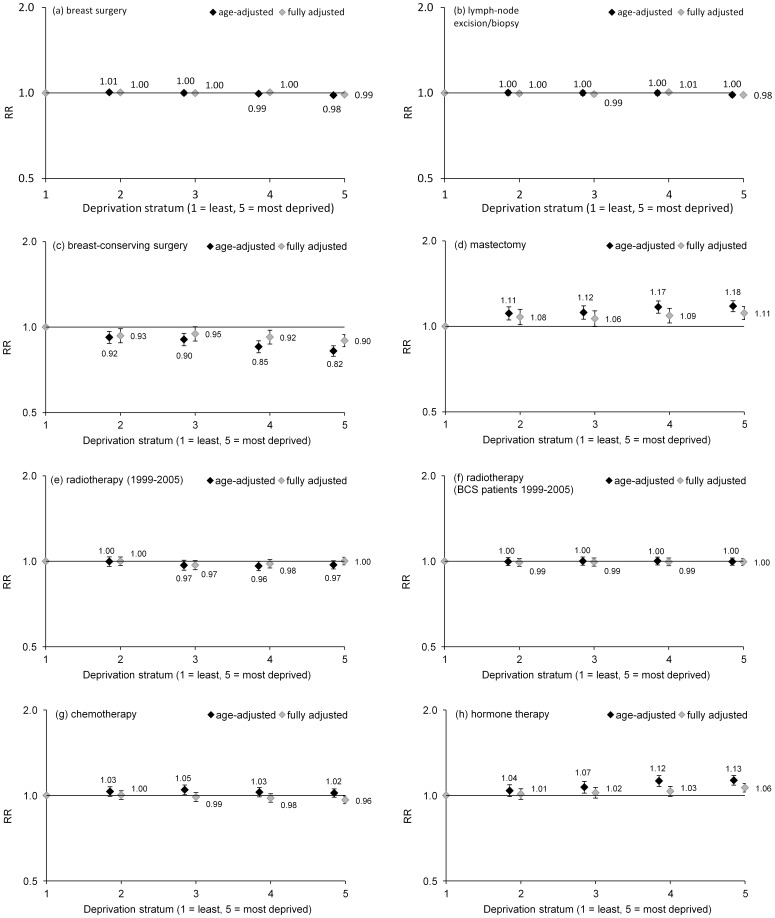
Relative risks for breast cancer treatment by deprivation stratum, Ireland, 1999–2008: age-adjusted (black symbols) and fully adjusted (grey). Adjusted for diagnosis cohort, age-group, HSE area of residence, T, N and M categories of stage, tumour grade, method of presentation, smoking status, marital status (except hormone therapy), tumour morphology, hormone receptor status and HER2 status (except radiotherapy), also surgery type (for radiotherapy only).

See Figure 2 for treatment variation by deprivation stratum.

*P<0.05, **P<0.01, ***P<0.001.

#Age-adjusted relative risks for stratum 5 v 1, and for average per-unit change across deprivation strata 1–5, exclude unknown values for relevant patient, tumour and treatment variables. ^a^Fuller details of tumour and patient characteristics (e.g. finer age-groups) were used in statistical modeling of survival. ^b^European age-standa*r*dized rate (per 100,0000 women per year). ^c^Age-standardized incidence ratio. ^d^Cases used for survival analysis exclude: diagnosis age <15 or >99; cancers preceded by another breast cancer or other malignant cancer (excluding non-melanoma skin cancers); death-certificate-only, autopsy-only and other cases with diagnosis = death date. ^e^Cases with known comorbidity status (based on hospital patient linkage) 2002–2008: 13039 (82.4%) of 2002–2008 total 15827. ^f^Deprivation stratum was unknown for 1826 cases (not shown separately).

After relevant exclusions (see Methods), data on 21356 women (3906 breast cancer deaths) were retained for survival analysis for the years 1999–2008. Deprivation status was known for 19694 women (92%), and 26% of known-status women were resident in the least deprived areas (stratum 5), 28% in the most deprived areas (Table 1). Analyses for 1999–2008 were based on 99072 person-years of follow-up, with a median follow-up time of 4.2 years; 42% of patients had at least 5 and 24% at least 7 years of follow-up. For assessment of time-trends, survival patterns by deprivation were examined for a further 7930 cases diagnosed during 1994–1998 (comparisons restricted to five-year follow-up).

### Variation of patient and tumour characteristics by deprivation status

Women from the most deprived strata were less likely to be aged under 50 (P<0.001) and more likely to be aged 65 or over at diagnosis (P<0.001), compared with the least deprived stratum (Table 1). All further analyses of case characteristics, treatment and survival by deprivation stratum have been adjusted for age (five age-groups). Patients from more deprived strata were less likely to be resident in Dublin/Mid-Leinster and more likely to be resident in the South and West regions (P<0.001). They were also more likely to be smokers and unmarried (P<0.001). In a subsample analysis of 13039 patients diagnosed during 2002–2008, for whom linked public hospital records were available, patients from more deprived strata were more likely to have significant comorbid conditions (Charlson Index 1 or 2, P<0.001).

Patients from deprived strata were less likely to be diagnosed with Stage I or T1 (P<0.001) or grade 1 breast cancer (P<0.01), and more likely to be diagnosed with Stage III (P<0.01), stage IV or T4 (P<0.001), node-positive or grade 3–4 disease (P<0.01). Cases from deprived strata were also less likely to be HER2-negative (P<0.05), more likely to be hormone receptor-negative (P<0.001), and less likely to involve lobular adenocarcinoma (P = 0.06 for stratum 5 vs. 1, P = 0.03 for trend). Patients from deprived strata were less likely to have presented via screening (P<0.001) (Table 1).

### Treatment variation by deprivation status

There was no association between deprivation and use of lymph-node biopsy/excision (Figure 2b) or overall tumour-directed treatment. Women from more deprived strata were slightly less likely to have tumour-directed breast surgery (age-adjusted relative risk 0.98 for stratum 5 v 1, 95% CI 0.97–0.99, P<0.05) (Figure 2a). Differences were more marked for type of surgery: use of breast-conserving surgery (BCS) was substantially lower (RR 0.82, 95% CI 0.79–0.86, P<0.001) and use of mastectomy greater (RR 1.18, 1.13–1.23, P<0.01) in the most deprived group (Figure 2c–d).

Radiotherapy use varied little by deprivation stratum, overall or following BCS (Figure 2e–f). This was confirmed by a sub-analysis excluding the years 2006–2008 when radiotherapy data were known to be slightly incomplete for private-hospital patients (NCR unpublished). But patients from the most deprived stratum started radiotherapy later (P<0.001) in relation to median treatment time (age-adjusted RR for treatment ≥ median time = 1.12, 95% CI 1.07–1.17, P<0.001; fully adjusted RR 1.06, 1.02–1.10, P = 0.008).

Chemotherapy use showed no significant trends by deprivation (Figure 2 g). Recorded hormone therapy use was highest in those from deprived backgrounds (both overall, RR 1.13 for stratum 5, 95% CI 1.09–1.18, and among hormone receptor-positive patients, P<0.001) (Figure 2 h).

Adjustment for other patient and tumour characteristics substantially weakened most deprivation-related trends in treatment, but there remained significant variation in use of BCS and mastectomy and, to a lesser extent, hormonal treatment (Figure 2). The fully adjusted trend for chemotherapy suggested a weak but steady trend towards lower use in more deprived groups (Figure 2 g).

### Survival variation by deprivation stratum: descriptive statistics and age-adjusted models

**Figure 3:**
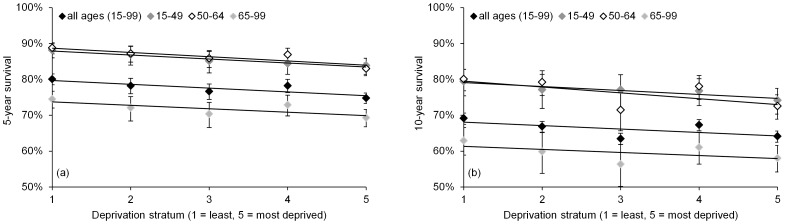
Five-year (a) and ten-year (b) cause-specific survival of female breast cancer patients, Ireland, 1999–2008: by deprivation stratum, all ages (age-standardized) and by diagnosis age.

**Figure 4:**
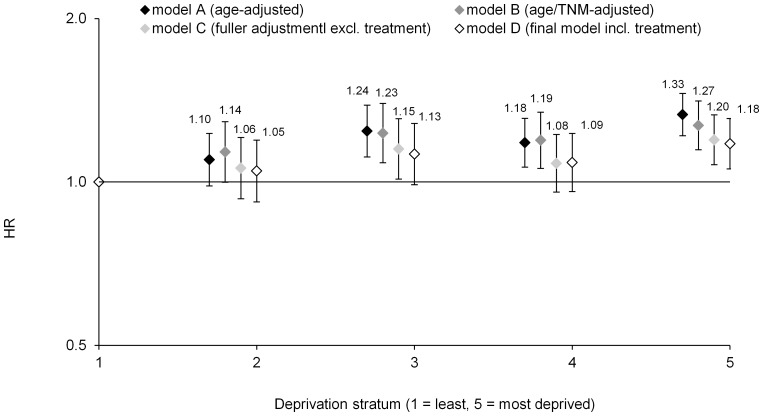
Hazard ratios for cause-specific mortality by deprivation stratum for female breast cancer patients, Ireland, 1999–2008: comparison of models from A (age-stratified) to D (fully adjusted). See Table 2 for further details of the models.

Cause-specific survival during 1999–2008 showed a strong inverse relationship with area-based deprivation scores: age-standardized five-year survival ranged from 80.0% in stratum 1 (least deprived) to 74.7% in stratum 5 (most deprived) (Figure 3a), ten-year survival from 69.1% to 64.1% (Figure 3b). Patients from the most deprived stratum were 33% more likely to die from their cancer than patients from the least deprived stratum (age-adjusted hazard ratio = 1.33, 95% CI 1.21–1.45: Table 2 and Figure 4, model A).

**Table 2:** Hazard ratios for cause-specific mortality by deprivation stratum for female breast cancer patients, Ireland, 1999–2008.

	Hazard ratio by deprivation stratum (1 = least, 5 = most deprived)					
	1	2	3	4	5	1–5 unit
*Model A*						
age-stratified^a^	1.00	1.10	***1.24	**1.18	***1.33	***1.07
		0.98–1.22	1.11–1.38	1.06–1.31	1.21–1.45	1.04–1.09
P		0.097	<0.001	0.002	<0.001	<0.001
*Model B*						
age/TNM-stratified^ab^	1.00	1.14	**1.23	**1.19	***1.27	***1.05
		0.99–1.29	1.08–1.39	1.05–1.34	1.14–1.40	1.02–1.07
P		0.052	0.001	0.004	<0.001	<0.001
*Model C*						
Fuller adjustment excl treatment^c^	1.00	1.06	*1.15	1.08	1.20	1.04
		0.94–1.19	1.02–1.30	0.99–1.18	0.98–1.46	1.00–1.08
P		0.336	0.026	0.082	0.079	0.082
*Model D*						
Final model incl treatment^d^	1.00	1.07	1.15	*1.09	1.18	1.04
		0.94–1.21	1.00–1.32	1.00–1.19	0.97–1.43	0.99–1.08
P		0.300	0.057	0.049	0.093	0.089

(See Table 2 for fuller details of Model C.).

a *Model A:* stratified by age (to allow for non-proportional hazards).

b *Model B:* as model A but also stratified for T, N and M categories of stage.

c *Model C:* as model B but also stratified for tumour grade, and adjusted for method of presentation, smoking status, region of residence, diagnosis year, tumour morphology and hormone receptor status; standard errors adjusted for clustering by region of residence; marital status and HER2 status excluded as they did not significantly contribute to model-fit); Charlson Index of co-morbidity (available for 82% of cases 2002–2008) excluded as it did not contribute significantly to model-fit.

d *Model D:* as model C but also adjusted for treatments within 12 months after diagnosis: breast surgery (mastectomy, BCS, or none); radiotherapy (month of treatment, or none); chemotherapy & other non-hormonal medical oncology (month of treatment, or none); hormone therapy (< = median month to treatment, yes>median, none); any tumour-directed treatment (yes, no); regional lymph-node excision/biopsy (yes/no). Time to surgical treatment or to first tumour-directed treatment did not improve model fit further.

* P<0.05, ** P<0.01, *** P<0.001.

### Survival variation by deprivation stratum: multivariate models

We evaluated the survival/deprivation relationship further by constructing a series of multivariate models (Table 2 and Figure 4, models B–D). For the period 1999–2008, adjustment (stratification) for TNM and age reduced the hazard ratio for deprivation stratum 5 from 1.33 to 1.27 (95% CI 1.14–1.40) (model B). This is, at face-value, equivalent to stage ‘explaining’ about 20% of the deprivation-related disparity (compared with the baseline age-adjusted model), although an such interpretation may not strictly be valid [40]. Adjustment for region of residence, smoking status, diagnosis year, grade, method of presentation, tumour morphology and hormone receptor status further reduced the HR for stratum 5 to 1.20 (0.98–1.46) (model C), about a 40% reduction of the age-adjusted disparity. Marital status and HER2 status, which did not contribute significantly to model-fit, were excluded. A sub-analysis of 2002–2008 data (for which comorbidity data were available) indicated that the Charlson Index of comorbidity likewise did not contribute significantly to model-fit, nor did its inclusion alter HR estimates compared with simpler model.

The final model included, in addition to patient and tumour factors, summary data on treatment. Inclusion of treatment variables reduced the HR for stratum 5 only slightly, to 1.18 (0.97–1.43) (Table 2 and Figure 4, model D), about a 45% reduction of the survival disparity compared with the age-adjusted model.

Although we found some evidence from unadjusted and age-adjusted models of heterogeneity of the influence of deprivation across patient subgroups defined by age, region and some other variables (see ‘*Survival variation by deprivation stratum: heterogeneity by patient subgroup*’ below), inclusion of interaction between deprivation and the variables involved did not significantly improve the fit of models C and D, therefore interaction terms were not included in the final models.

### Summary of other patient and tumour factors influencing survival

Mortality hazard ratios associated with other patient and tumour factors adjusted for in model C (Table 2) are summarized in Table 3. Breast cancer mortality was significantly higher for patients resident in regions other than Dublin/Mid-Leinster; current smokers and patients of unknown smoking status, compared with non-smokers; cases detected symptomatically or incidentally, compared with screen-detected cases; and cases of unspecified histological type, compared with ductal adenocarcinoma. Mortality was significantly lower for cases with positive or unknown oestrogen or progesterone receptor status, compared with receptor-negative cases. Diagnosis year contributed significantly to model-fit, but improvements in survival in more recent years were, in general, not statistically significant at the annual scale used in this model.

**Table 3:** Further details of model C (Table 2): other patient and tumour factors influencing breast cancer survival (in a model assessing the influence of area-based deprivation status), 1999–2008.

	Hazard ratio	95% CI	P
15–44 diagnosis age	(1.00)	–	
45–54	(0.99)	0.92–1.07	0.838
55–64	***(1.23)	1.12–1.36	<0.001
65–74	***(1.53)	1.43–1.64	<0.001
75–99	***(2.25)	2.07–2.46	<0.001
T0	(0.29)	0.02–4.55	0.378
Tis	(0.68)	0.17–2.71	0.580
T1 (NOS) (< = 2 cm)	(0.93)	0.75–1.15	0.497
T1m (< = 0.1 cm)	(0.21)	0.04–1.22	0.082
T1a (>0.1<-0.5 cm)	***(0.39)	0.30–0.50	<0.001
T1b (>0.5< = 1.0 cm)	*(0.74)	0.55–0.98	0.039
T1c (>1.0<2.0 cm)	(1.00)	–	
T2 (>2.0<5.0 cm)	***(1.54)	1.42–1.67	<0.001
T3 (>5.0 cm)	***(2.36)	2.00–2.79	<0.001
T4 (NOS)	***(2.72)	2.22–3.33	<0.001
T4a (chest wall)	***(3.23)	2.68–3.90	<0.001
T4b (skin)	***(2.83)	2.81–3.462	<0.001
T4c (both)	***(4.13)	2.89–5.90	<0.001
T4d (inflammatory)	***(3.27)	2.65–4.04	<0.001
T not applicable	**(8.43)	2.27–31.4	0.001
TX	***(1.91)	1.74–2.09	<0.001
N0	(1.00)	–	
N1	***(2.09)	2.01–2.17	<0.001
N2	***(2.67)	2.23–3.21	<0.001
N3	***(4.03)	3.51–4.63	<0.001
NX	***(2.43)	2.29–2.58	<0.001
M0	(1.00)	–	
M1	***(4.92)	4.69–5.16	<0.001
MX	**(1.13)	1.10–1.16	<0.001
grade 1	(1.00)	–	
grade 2	***(1.72)	1.41–2.11	<0.001
grade 3-4	***(2.59)	2.34–2.86	<0.001
grade unknown	***(2.20)	2.07–2.34	<0.001
1999 diagnosis year	1.00	–	
2000	1.09	0.99–1.19	0.071
2001	0.96	0.79–1.16	0.649
2002	1.07	0.98–1.17	0.126
2003	0.93	0.75–1.14	0.461
2004	1.02	0.78–1.35	0.862
2005	**0.86	0.77–0.96	0.006
2006	*0.89	0.81–0.99	0.025
2007	0.90	0.80–1.01	0.072
2008	0.79	0.61–1.01	0.056
Dublin/Mid-Leinster	1.00	–	
Dublin/North-East	***(1.20)	1.18–1.23	<0.001
South	***(1.17)	1.15–1.19	<0.001
West	***(1.24)	1.20–1.28	<0.001
screen-detected	1.00	–	
incidental	***2.12	1.97–2.28	<0.001
symptoms	***1.76	1.56–1.97	<0.001
unknown presentation	***1.28	1.16–1.41	<0.001
non-smoker	1.00	–	
ex-smoker	1.09	0.96–1.23	0.191
smoker	***1.15	1.08–1.23	<0.001
unknown	**1.20	1.08–1.35	0.001
ductal adenocarcinoma	1.00	–	
lobular adenocarcinoma	0.94	0.88–1.02	0.141
other adenocarcinoma	0.83	0.70–1.00	0.050
other carcinoma	0.97	0.81–1.15	0.715
carcinoma NOS	1.10	1.00–1.22	0.059
cancer NOS	***2.06	1.74–2.45	<0.001
sarcoma	0.91	0.18–4.70	0.915
malignant phyllodes tumour	0.49	0.25–0.96	0.037
hormone receptor negative	1.00	–	
hormone receptor positive	***0.50	0.45–0.56	<0.001
receptor status unknown	**0.72	0.59–0.88	0.001

Only factors which contributed significantly to model-fit are shown^a^; hazard ratios for age, TNM and grade are not shown, as the model shown in parentheses for age, TNM and grade are from simpler, adjusted but unstratified models (the more definitive model was stratified for those variables).

a Marital status and HER2 status excluded as they did not significantly contribute to model-fit); Charlson Index of co-morbidity (available for 82% of cases 2002–2008) excluded as it did not contribute significantly to model-fit (in 2002–2008 sub-analysis); standard errors adjusted for clustering by region of residence. Models adjusted for (or stratified by) coarser groupings of variables tabulated had a poorer fit to the data.

* P<0.05, ** P<0.01, *** P<0.001.

Model C was stratified for age, TNM and grade, because these variables were found to show non-proportional hazards, thus definitive hazard ratio estimates were not available. However, hazard ratios from an equivalent but simpler model (adjusted but unstratified) are shown in parentheses in Table 3. These indicate significantly poorer cause-specific survival in age-groups 55–64 and older, compared with 15–44; T2–T4 and T-unknown cases, compared with T1c; N1-3 and N-unknown cases, compared with N0; M1 and M-unknown cases, compared with explicit M0; and grade 2–4 and grade-unknown cases, compared with grade 1. Survival was significantly better for T1a and T1b cases, compared with T1c.

### Survival variation by deprivation stratum: time-trends

**Figure 5:**
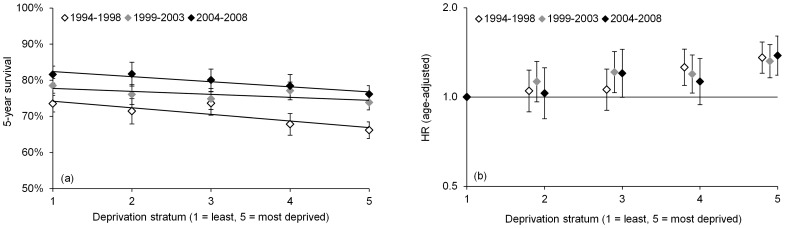
Five-year cause-specific survival (age-standardized) and age-adjusted mortality hazard ratios for female breast cancer patients, Ireland, 1994–2008: by deprivation stratum and diagnosis cohort.

**Figure 6:**
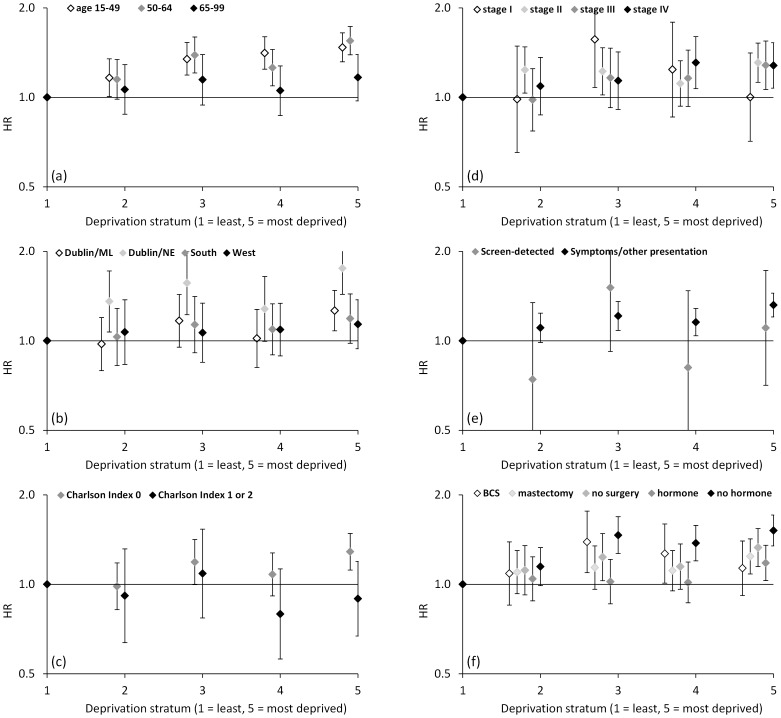
Hazard ratios for breast cancer mortality by deprivation stratum, Ireland, 1999–2008: by (a) age of diagnosis and (age-adjusted) by (b) region of residence, (c) Charlson Index of comorbidity, (d) TNM 5^th^-edition stage, (e) method of presentation, and (f) surgical and hormonal treatment. Trends by deprivation were less heterogeneous for other subgroups.

To explore our findings further, we also examined possible changes over time in the deprivation-related trend (Figure 5), and variation of the deprivation-related trend across different patient subgroups (Figure 6).

Comparisons of age-standardized five-year survival for three patients cohorts, including 1994–1998 (preceding the main study period), indicate that all deprivation strata have benefitted from improvements in survival from 1994 to 2008 (Figure 5a). However, socioeconomic disparities have persisted, with five-year survival in the most deprived group consistently 5 to 7 percentage points lower than in the least deprived group in all three cohorts. Age-adjusted Cox models confirm that all three periods showed similar deprivation trends, with a RR for deprivation stratum 5 (*vs*. 1) of 1.08 for 1994–1998 (95% CI 1.05–1.11), 1.07 for 1999–2003 (1.03–1.10) and 1.08 for 2004–2008 (1.04–1.12) (P = 0.76, P = 0.67 and P = 0.88 for 1994–1998 v. 1999–2003 comparison, 1999–2003 v. 2004–2008, and 1994–1998 v. 2004–2008, respectively, or P = 0.44, P = 0.58 and P = 0.90 based on average trends across strata 1–5) (Figure 5b). Fuller comparisons, adjusting for other tumour characteristics and treatment, were not attempted, because availability of these data varied substantially between cohorts.

### Survival variation by deprivation stratum: heterogeneity by patient subgroup

For the period 1999–2008, formal comparisons were made of age-adjusted HRs (for deprivation) between patient subgroups defined on the basis of a range of patient, tumour and treatment factors, and of unadjusted HRs between age-groups. Broadly similar trends were seen across most subgroups, but the influence of deprivation on survival differed by diagnosis age, region of residence, and comorbidity (Figure 6a–c), and by receipt of hormonal treatment and timing of radiotherapy and chemotherapy (Figure 6f). Less clear-cut heterogeneity was seen for some other factors including stage (Figure 6d-f and summary below). However, the apparent heterogeneity seen in these sub-analyses did not take account of other variables (apart from age), and no significant interactions with deprivation were evident when testing multivariate models (see ‘*Survival variation by deprivation stratum: multivariate models*’ above).

Patients diagnosed at ages 15–49 and 50–64 showed a stronger trend towards higher breast cancer mortality in more deprived groups (HRs 1.47 [95% CI 1.20–1.80] and 1.55 [1.32–1.82], respectively, for stratum 5), compared with a significant but weaker trend for ages 65–99 (HR = 1.17 [1.03–1.33] for stratum 5; P = 0.007 for difference from age 50–64) (Figure 6a). The same pattern by age was also evident if analysis was restricted to symptomatically detected cases: at age 15–49, HR for stratum 5 = 1.49 (95% CI 1.20–1.86); at age 50–64, HR 1.45 (1.21–1.74); at age 65–99, HR 1.15 (0.99–1.32) (P = 0.007 for difference from age 50–64) (data not graphed).

By region of residence, only Dublin/Mid-Leinster and Dublin/North-East showed statistically significant deprivation effects (HRs 1.26 [95% CI 1.08–1.48] and 1.75 [1.43–2.14], respectively, for stratum 5), and the effect was stronger in Dublin/North-East than in the other three regions (P<0.01 for differences) (Figure 6b).

Patients without comorbidities had significantly higher mortality in the most deprived stratum (HR = 1.29 [95% CI 1.12–1.48], 2002–2008 sub-analysis), but those with comorbidities (Charlson Index 1–2) showed no clear deprivation trend (HR = 0.89 [0.67–1.12]; P = 0.03 for difference) (Figure 6c). Heterogeneity was not significant by stage at diagnosis, but stage I patients showed no clear mortality trend by deprivation (HR 1.00 [0.71–1.41] for stratum 5) in contrast to stages II, III and IV (HRs 1.31 [1.12–1.52], 1.28 [1.06–1.52]_and1.28 [1.07–1.53], respectively; P>0.16 for differences) (Figure 6d). Likewise, there was no significant heterogeneity in trend by screening status, but no clear deprivation trend was evident among screen-detected cases, unlike symptomatic and other cases (HR 1.32 [1.20–1.45] for stratum 5) (Figure 6e).

Patients without recorded hormonal treatment showed a stronger mortality trend by deprivation (HR 1.52 [95% CI 1.35–1.71] for stratum 5) than those with hormonal treatment (HR 1.18 [1.03–1.35] for stratum 5; P = 0.007 for difference) (Figure 6f). The trend did not vary by radiotherapy or chemotherapy status (Figure 6f) but was perhaps more marked among patients who started these treatments sooner after diagnosis (no significant trends for patient treated later than median time after diagnosis; P = 0.09 and P = 0.07 for differences, respectively). Women who had breast-conserving surgery showed a non-significant deprivation trend (HR 1.13 [0.92–1.40] for stratum 5), while those who had mastectomy or no surgery showed significant trends (HRs 1.24 [1.08–1.42] and 1.33 [1.15–1.54], respectively; P>0.10 for difference from BCS) (Figure 6f).

## Discussion

Irish women who were diagnosed with breast cancer during 1999–2008 were 33% more likely to die of their cancer if they lived in the most deprived areas, compared with women in the least deprived areas, having adjusted for age. This association was widespread across patient subgroups but appeared to be more pronounced in some subgroups, for example women under 65 and the Dublin/North-East and Dublin/Mid-Leinster regions. Survival steadily improved over the period 1994 to 2008, but disparities by socioeconomic status were evident throughout and had not diminished by the most recent period.

Using available information on patient and tumour characteristics and treatment, we were able to ‘explain’ no more than half of the disparity in breast cancer survival between the most and least deprived groups (fully adjusted mortality hazard 18% higher in the most deprived group, although this was no longer statistically significant). This suggests there may be other factors, mechanisms or complexities involved. However, in addition to random error, there are methodological concerns about the validity of using adjusted models to apportion the contribution of individual (mediating) factors in epidemiological analyses [12], [40]. This caveat should be borne in mind regarding the conclusions of other studies discussed below.

The incidence of breast cancer in Ireland is higher among more affluent women, consistent with reports from other countries and linked to socioeconomic differences in demographic and reproductive factors (notably parity and age at first birth) and, to some extent, participation in screening [19], [26]. Increasing incidence in Ireland in more recent years can be at least partly explained by the introduction of population-based screening and demographic changes [1]. Breast cancer mortality is falling in most countries, including Ireland, but not all sectors of society are benefitting equally. Many authors have explored the role of socioeconomic disparity in breast cancer survival, at individual or area level, with broadly similar findings to ours. For example, in the US age/year-adjusted cancer-specific mortality was 19% higher for breast cancer patients from the lowest compared with the highest area-based socioeconomic group [18]. In contrast to our study, the excess hazard was largely removed (HR = 1.03) by adjusting for stage, treatment and race. In eastern England, relative hazards of 1.23 and 1.22 (after adjustment for age and stage) were reported for breast cancer mortality among women in the most deprived areas and lowest social class, respectively [15]. In the Thames region of England, a relative hazard of 1.35 was noted (after adjustment for stage, morphology and treatment) for the most versus least deprived categories, with a deprivation gap of >10% for five-year survival [21]). A smaller disparity was noted in northern England (HR 1.11 age-adjusted, 1.09 age/stage-adjusted) [17]. The majority of such studies, like ours, have not been able to fully account for the deprivation gaps seen for breast cancer.

Any discussion of the impact of socioeconomic disparities on health and on breast cancer survival raises a complex array of factors explored by many authors [3], [23], [26], [41]. Besides material, educational and psychosocial factors, early life exposures to the effects of poverty, and stressors associated with lack of control over work, potentially contribute to disparities in breast cancer survival, along with cultural and genetic factors [41]. These factors are often not available in clinical or cancer registry datasets, and studies attempting to explain survival disparities have generally focused on factors or intermediate end-points closer to diagnosis or treatment, as discussed below.

### Stage

Stage at diagnosis is an important predictor of breast cancer survival [42], and we found that Irish women from deprived areas were more likely to be diagnosed at advanced stages. Survival disparities across deprivation strata appeared to be more marked in stages II-IV, and stage appeared to account for a more substantial proportion of the survival disparities seen than other factors. However, our study was unable to assess delays in diagnosis directly because we lacked comprehensive data on referral or consultation dates or on duration of pre-diagnosis symptoms.

Many other studies have found an association between deprivation and late presentation with breast cancer, but the association and its contribution to survival disparities vary. One Scottish study found no variation in stage by socioeconomic deprivation and concluded that it could not account for survival disparities [20]. A study in eastern England concluded that stage accounted for 28% of the effect of individual-level social class, but none of the effect of area-level deprivation category, on survival [15]. In northern England, breast cancer patients from deprived areas were more likely to have stage III or IV disease, but adjustment for stage reduced deprivation-related survival disparities only slightly [17]. In the Netherlands, patients of lower socioeconomic status had more advanced stage and, among non-screen-detected cases, stage explained about half of the socioeconomic survival disparities seen [12].

### Grade, morphology, tumour receptors

Other aspects of tumour aggressiveness also showed some relationship to deprivation in our study. Patients from poorer areas were more likely to present with high grade or hormone receptor-negative disease, and less likely to present with lobular adenocarcinoma. Grade, hormone receptor status and histological subtype all contributed to our final model of survival by deprivation status, but their explanatory role appeared to be less than that of stage.

In Scotland, one study found no variation in grade or oestrogen receptor status by deprivation category [20], while another found a higher proportion of oestrogen receptor-positive cases among affluent patients but suggested this only partly explained the survival disparity seen [14]. The review by Wood et al. [26] likewise notes that variations of such “biological” characteristics by socio-economic status are not always consistent, nor are the reasons for such variations. Although such characteristics may not be directly associated with delays in diagnosis, an indirect association can be inferred from studies comparing screen-detected with symptomatic tumours, for example a higher proportion of hormone receptor-positive tumours among screen-detected cases [43]. Likewise, in our study, grade 3–4 tumours accounted for 24.5% of screen-detected cases (compared with 43.9% of symptomatic cases), hormone receptor-negative cases 9.2% (18.8%), and lobular adenocarcinomas 17.7% (14.5%), excluding “unknown” categories. But possible associations between lifestyle factors and tumour biology [26] cannot be ruled out.

### Screening

Population-based mammographic screening was introduced in Ireland around 2000 for women aged 50–64, initially in Dublin and eastern parts of the country, then more widely. About 16% of Irish cases during 1999–2008 presented through screening, and women from more deprived backgrounds were less likely to present this way. No clear survival trend by deprivation was evident among screen-detected cases, unlike symptomatic cases, but numbers of cases (and deaths) were small. Adjusting for screening status did appear to reduce the survival disparities seen, apparently independently of its influence on stage, grade or treatment, but its influence seemed minor.

The potential contribution of mammographic screening to survival disparities is complex (even without considering lead-time bias i.e. over-estimation of the survival benefits of early diagnosis). For example, in the Netherlands survival and stage distribution of breast cancer patients improved after the introduction of population-based screening, but less so in the lower socioeconomic groups, and survival disparities actually widened compared with pre-screening years [4]. The authors suggested this was due to higher comorbidity and less optimal treatment among more disadvantaged patients rather than differences in screening attendance, but a further study acknowledged the survival disparities were at least partly related to screening attendance [12]. In England and Wales, in contrast, it was suggested that a narrowing of socioeconomic survival inequalities for breast cancer between 1973 and 2004 may have reflected progressive introduction of ‘new’ interventions (notably screening) that improved survival to a lesser extent than earlier interventions (adjuvant chemotherapy and endocrine therapy) [30]. No obvious change in survival disparities is, as yet, evident among Irish patients, but population-based screening was not widely available until late in the study period.

### Comorbidity

Of patients we could match to hospital inpatient records, 10.9% had significant comorbid conditions recorded, highest (13.5%) among women from the lowest deprivation stratum. This subgroup showed no clear trend in survival disparity by deprivation, in contrast to women without comorbid conditions, a finding we cannot explain. Incorporating comorbidity in our survival models did not improve model-fit or modify the survival disparities. Other studies have noted that breast cancer patients of lower socioeconomic status are more likely to have comorbid conditions [2], [4], [29], and some have suggested that this contributed to breast cancer survival disparities. For example, in the Netherlands, comorbidity was estimated to account for 23% of the survival disparity among screen-detected cases [12]. This impact on cancer-specific survival is thought to relate in part to less aggressive treatment of patients with comorbidities but can also be independent of treatment [29]. Possibly the lack of a clear explanatory role of comorbidity in our study could reflect insufficient discriminatory power if some relevant conditions were not recorded in hospital data – for example, if low-level or undiagnosed comorbidities were more frequent in poorer patients. Also, the role of comorbidity could be less important for breast cancer than for cancers with an older age-profile, and further analyses focused on older women might be informative.

### Smoking

Irish patients from the most deprived areas were more likely to be smokers, and smokers had poorer cause-specific survival in a fully adjusted model. Smoking appeared to be only a minor contributor to the survival disparities seen (which were also evident among non-smokers), but we lacked information on detailed patterns of tobacco consumption. The potential contribution of smoking to breast cancer survival disparities is acknowledged by some other studies [4], but few studies seem to have explored its role in this context.

### Age at diagnosis

Absolute survival differences between deprivation strata were similar across age-groups, but the deprivation-related trend in mortality hazard ratios was significantly less marked among women aged 65 and over. One possible explanation might be that the trend for older women was clouded by substantial age-related treatment disparities in Ireland [1]. Another is that screening-mediated disparities were more likely in younger women, but even among symptomatic cases deprivation-related survival disparity was significantly lower in the age 65+ group. It has also been suggested that deprivation indicators developed for general or younger populations may not always be appropriate for studies of health inequalities in older populations [44]. But a Scottish study noted similar survival disparities in younger and older breast cancer patients [14].

### Geographic factors

Deprivation-related survival disparities were greatest among women from the more urbanized regions, Dublin/North-East and Dublin/Mid-Leinster. Population-based screening was introduced in these regions earlier than elsewhere, and they contain most of the academic medical centres in Ireland. In a fully adjusted model, survival was significantly higher in Dublin/Mid-Leinster than in any of the other regions. Treatment of Irish breast cancer patients is known to vary by region [1], and in Scotland regional differences in survival were considered to be compatible with regional variation in adjuvant therapy use [6]. But it may be that not all sectors of society benefit equally from such regional advantages. There is also evidence that area-based deprivation indexes, in Ireland and elsewhere, may provide a less good measure of socioeconomic status in rural areas [45].

### Treatment

We found no significant variation for overall treatment and only minor variation for overall breast surgery and radiotherapy by deprivation status. But breast-conserving surgery (BCS) use was substantially lower and mastectomy higher among more deprived groups, even after adjustment for stage and other factors, although radiotherapy use after BCS showed no trend. Chemotherapy use was slightly lower among more deprived groups, in a fully adjusted model. Use of hormone therapy appeared to be higher in more deprived groups, but we could not exclude this being an artefact of data completeness varying by deprivation status. Survival disparities appeared to be stronger among patients with no recorded hormonal treatment, and those having mastectomy or no breast surgery compared with BCS (P>0.10). One possible explanation might be that there was less potential for mortality differentials among ‘lower risk’ patients (e.g., those having BCS or hormonal treatment).

Only radiotherapy showed evidence of later treatment among patients from deprived backgrounds. In general, deprivation-related survival disparities were similar for patients whose treatment started at different times after diagnosis, but the disparity appeared to be greater among patients who received radiotherapy or chemotherapy sooner. It may be that women diagnosed at a later stage are more likely to be treated rapidly, and the literature in general does not support the idea that delays by the healthcare provider explain the survival disparities in breast cancer [26].

In the final model of survival disparities, adjustment for treatment improved model-fit but only slightly reduced the hazard ratios associated with deprivation. However, we lacked information on some aspects of treatment such as patients’ compliance, reasons for non-treatment or specific chemotherapy regimens which potentially could also have contributed to survival disparities.

In northern England, breast cancer patients from more deprived areas were less likely to have surgery, breast-conserving surgery or radiotherapy, after adjustment for age and stage, and more likely to have appointment or treatment delays >14 days, but use of chemotherapy and hormone therapy was not influenced by deprivation [17]. Use of breast-conserving surgery was also less frequent in more deprived groups in a Scottish study [14]. In the Netherlands, use of adjuvant chemotherapy was lower among stage II patients from lower socioeconomic groups, and disparities in chemotherapy use may have contributed to widening survival disparities over time [4]. Many other studies have demonstrated treatment differences by socioeconomic status, but with mixed conclusions as to the contribution of treatment to survival disparities [18], [23].

Irish health care is two-tier, with many patients reliant on public care but many others with insurance policies providing access to private care. However, the magnitude of deprivation-related survival disparities for breast cancer is broadly similar in many studies, even in countries with (theoretically) more equitable access to treatment than Ireland.

### Factors not measured

At individual patient level, we had limited information on lifestyle factors and none on educational attainment. In a study where both individual-level and community-level measures of educational disparity were collected, both measures influenced breast cancer survival but the community measure remained an independent predictor (HR = 1.39) even after controlling for individual-level education and other confounders [9].

There is some, but not always consistent, international evidence that alcohol consumption is higher in populations of lower socioeconomic status and that this contributes to socioeconomic gradients in incidence of certain cancers [23]. Alcohol is an established risk factor for breast cancer incidence [46], and rising alcohol consumption among Irish women [47] may contribute to the rising incidence of breast cancer here. Alcohol’s role in breast cancer mortality after diagnosis is less clear [48], with light drinking being protective but heavy drinking a risk factor. Alcohol may contribute to higher cancer-specific mortality in more deprived patients, and perhaps account for some of the ‘unexplained’ survival disparities seen in our study.

Obesity increases the risk of both breast cancer incidence and mortality in post-menopausal women [49]. Height, weight and dietary factors are not routinely measured in Irish hospitals, thus unavailable in NCR data, but obesity is higher in women from manual social classes in England [50] and may account for a further proportion of the survival disparities we noted.

### ‘Avoidable’ deaths

In England, an estimated 25% of excess deaths due to breast cancer (within three years of diagnosis) would be avoided if relative survival for all patients matched survival for the most affluent group [31]. Equivalent figures for Ireland are not readily calculated, because our outcome was cause-specific survival, but it seems likely that broadly similar proportions would apply here, given the magnitude of the survival disparities we have described.

### Strengths and limitations of the study

Our study benefited from the availability of data from population-based national coverage of breast cancer cases in Ireland, including comprehensive data (by international cancer registry standards) on patient and tumour factors and treatments. But we lacked individual or small-area data on some relevant factors, such as obesity, alcohol consumption and health-seeking behaviours that may contribute survival disparities in breast cancer survival. Good basic information was available on treatment, but details of treatment decision-making or compliance were not available in the cancer registry dataset.

## Conclusions

Our analysis shows that socioeconomic deprivation is a major predictor of mortality among Irish breast cancer patients, and that a range of patient, tumour and treatment factors (notably stage, grade, screening status, smoking status and comorbidity) varied by deprivation status in a direction consistent with disparities in survival. But we were unable to account for more than about half of the disparities seen. Future studies may be able to evaluate a fuller array of risk factors, including personal-level as well as community-level variables, to produce a better understanding of the causes of survival disparity. Nevertheless, policy implications of our findings are clear. Survival disparities need to be reduced and this could be aided by targeting areas of known disparity, to encourage greater awareness of the importance of early detection, treatment compliance, lifestyle factors that may affect treatment response, and post-treatment check-ups, and to ensure that medical facilities can be accessed without undue financial or other barriers.
